# Effects of Outdoor Walking on Positive and Negative Affect: Nature Contact Makes a Big Difference

**DOI:** 10.3389/fnbeh.2022.901491

**Published:** 2022-06-03

**Authors:** Fabien D. Legrand, Philippe Jeandet, Fabien Beaumont, Guillaume Polidori

**Affiliations:** ^1^Laboratoire Cognition, Santé, Société, Université de Reims Champagne Ardenne, Reims, France; ^2^Laboratoire Résistances Induites et Bioprotection des Plantes, Université de Reims Champagne Ardenne, Reims, France; ^3^Laboratoire MATIMM, Université de Reims Champagne Ardenne, Reims, France

**Keywords:** green exercise, nature, positive affect, walking, nature relatedness, pollution

## Abstract

It has been consistently demonstrated that physical exercise is a cost-effective way to promote emotional well-being. However, the environment in which it takes place might amplify or mitigate this beneficial effect. The present study aimed at comparing the effects of walking in a natural or urban field setting on positive and negative affect. For this purpose, 150 students (46 female, 104 male; mean age: 20.2 years) were randomized into one of three groups: Green Walking (GW, *n* = 50), Urban Walking (UW, *n* = 50), or no-exercise (control; CTRL, *n* = 50). Positive and negative affect ratings were collected for each participant before and after walking (or before and after attending a class in the CTRL group). Exercise parameters (duration, intensity, weather conditions, group size) were identical in the GW and UW groups. The walking routes differed in terms of vegetation density, proximity of water, presence of traffic, and amount of asphalted surfaces. Participants in the GW and UW groups reported significant reductions in negative affect pre- to post walking. However, positive affect was increased only for participants in the GW group. This finding may have meaningful implications for mental health professionals who treat patients with significant emotional distress or mood instability. Several explanations are discussed as potential mechanisms for the more beneficial effect of Green walking, and presented as an important avenue for future research.

## Introduction

Emotions have traditionally been categorized on the basis of two broad dimensions, namely pleasantness, and arousal. Watson and Tellegen (1985) have suggested a 45-degree rotation of these axes to yield dimensions of positive and negative affect. While negative affect has been widely associated with the development of emotional disorders such as anxiety and mood disorders (Brown and Barlow, 2009), the role of positive affect in connection to these conditions remains relatively unexplored (Garland et al., 2010). In general, positive affect refers to pleasant emotional states, including joy, enthusiasm, energy, amusement, and alertness to cite a few. Of note, positive affect is not simply the opposite of negative affect, but rather an independent construct not always inversely correlated with negative affect (Gloria and Steinhardt, 2016), although this has historically been debated in the literature with other authors considering them to be bipolar ends of the same dimension (Ong et al., 2010).

Positive affect has been associated with many health-related benefits in clinical and non-clinical populations. For instance, research suggests it may increase stress resilience, as well as overall mental health and well-being (Lyubomirsky et al., 2005; Garland et al., 2010). In addition, positive affect has been associated with an improved functioning of the immune system (Diener and Seligman, 2002), which is of particular relevance in the current pandemic context. Likewise, preliminary research has established that explicitly targeting positive affect in mood and anxiety disorders treatment through therapeutic strategies or modules, such as well-being therapy (Barlow et al., 2017), positive psychotherapy (Hope et al., 2006), or an increase in the regulation of positive emotion (Eckeland et al., 2021) can lead to clinically significant improvements in symptoms.

Taken together, the scientific literature then clearly suggests that there is a benefit to increasing positive affect, but surprisingly, most of the psychotherapy interventions continue to focus on targeting negative affect. Accordingly, most studies of leading treatments for emotional disorders remain focused on evaluating outcomes of reduced symptoms of negative affectivity (Garland et al., 2010) rather than addressing positive affectivity. The question remains if and how existing interventions can modify positive affect even though it is not their central focus.

There is an extant literature suggesting that physical exercise might be well-suited for enhancing positive affect (Reed and Ones, 2006; Ekkekakis et al., 2011; Chan et al., 2019). Mood benefits (increased positive affect and/or decreased negative affect) have been consistently reported after a single bout of aerobic or anaerobic exercise at low or moderate intensities, and durations up to 35 min. However, individual responses to exercise can be moderated (either enhanced or lessened) by subtle factors of the physical or social environment, such as temperature, humidity, presence of mirrors, odors, etc. A relatively recent area of investigation has suggested that green exercise (that is, engaging in physical activity in the presence of Nature) could enhance the mood-boosting effect of exercise. According to Ulrich et al. (1991), this effect is linked to the fact that natural environments invoke feelings of interest and calm, thereby promoting positive affect. Another explanation recently offered by Baxter and Pelletier (2019) posits that human beings have a basic psychological need for nature relatedness. In congruence with the assumptions made by the Self-Determination Theory (SDT; Ryan and Deci, 2017), meeting this need would lead to vitality and well-being, as is the case with the other basic psychological needs (e.g., relatedness or competence). Several published studies have provided support for a positive relationship between natural environments and psychological well-being. For example, a large observational study including almost 95,000 adult participants revealed that residential greenness was associated with lower prevalence rates of major depressive disorder (Sarkar et al., 2018). Similarly, an experience-sampling study which examined the relationship between natural features of the environment and psychological well-being found that exposure to natural features (e.g., trees, birdsong) had an immediate beneficial effect on well-being lasting several hours (Bakolis et al., 2018). Hence, it is not surprising that many researchers are assuming that nature-based exercise environments promote greater improvements in psychological well-being than exercise in other environment types. In their systematic review, Thompson-Coon et al. (2011) confirmed the more beneficial effects of outdoor exercise on feelings of anger, confusion, tension, depression, enjoyment, energy, and revitalization. However, studies presented in this review have done little more than comparing indoor exercise with physical activity in any outdoor environment, which for the most part consisted of urban spaces. Only two studies have directly compared the psychological responses to exercise in a green vs. urban environment to date. In the study by Hartig et al. (2003), walking in oak-sycamore woodland nature settings increased positive affect and reduced anger whereas urban walks in a city retail and office space had the opposite effect. The second one is the study by Park et al. (2011) showing that tension, anxiety, fatigue, and confusion were decreased by a nature walk, but not by an urban walk of equivalent duration. Unfortunately, an important limitation of these two studies is that exercise intensity was not measured. The lack of experimental control for participants’ level of exertion is a significant issue given the well-established influence of this exercise parameter on affective responses to exercise (Ekkekakis et al., 2011). More specifically, an inverse relationship has been reported between exercise intensity and psychological well-being in the literature review by Ekkekakis et al. (2011), with positive affective changes occurring during and following exercise of both light and moderate intensities, but not for high or very high-intensity exercise.

Thus, the purpose of the present study was to more rigorously evaluate the hypothesis that Nature exposure has the possibility to enhance the affective benefits of outdoor physical activity. In this aim, we conducted a large randomized controlled trial comparing emotional changes following a 30-min outdoor exercise bout performed at a controlled and standardized level of intensity in either a natural countryside setting or an urban surrounding.

## Methods

### Study Design and Participants

We employed a randomized controlled design with three different groups: green walking (outdoor walking in connection to Nature), urban walking (outdoor walking in an urbanized area), or control (regular class time without any physical activity). Randomization was computer-generated through permuted blocks in a 1:1:1 ratio. Given the typical effect sizes (*ESs*) for exercise-induced affective changes (0.4–0.5, Reed and Ones, 2006), the *a priori* power analysis for the within-between interaction of an ANOVA with three independent groups and two measurement periods (pre and post) with the settings of *f* = 0.20, *α* = 0.05 (*p-*value), 1 − *β* = 0.95 (power) yielded a needed total sample size of 102 participants (G*POWER, Faul et al., 2007). A total of 150 undergraduate students from the Sport and Physical Education Department at a university in northeastern France were recruited. The mean age of this sample was 20.2 years (*SD* = 1.3) and 69.3% were male. The data were collected as part of a 2-h practical class, but at the time of data collection, the students were not aware of the exact hypotheses of the study. In particular, those belonging to the green and urban walking groups believed they were receiving an identical physical exercise stimulus. However, since experimenters knew which participants were in which group, our experiment can only be said to be a single-blinded study. Students’ academic timetables were carefully scrutinized to ensure that they did not exercise prior to our experiment. At the beginning of their visit, participants completed an informed consent and reported basic demographic information (age, sex).

### Instruments

Positive affect (PA) and negative affect (NA) were assessed using the Positive And Negative Affect Schedule (PANAS; Watson et al., 1988). This consists of 20 adjectives, each of which is rated on a 5-point scale from 1 (very slightly or not at all) to 5 (extremely). Ten items contribute to the positive affect scale (e.g., enthusiastic, excited, alert) and 10 to the negative affect scale (e.g., upset, afraid, irritable). Participants were asked to think about « how they were feeling right now» when completing the scales. Ratings were summed to generate positive and negative affect scores, each varying from 10 to 50.

### Procedure

**Figure 1:**
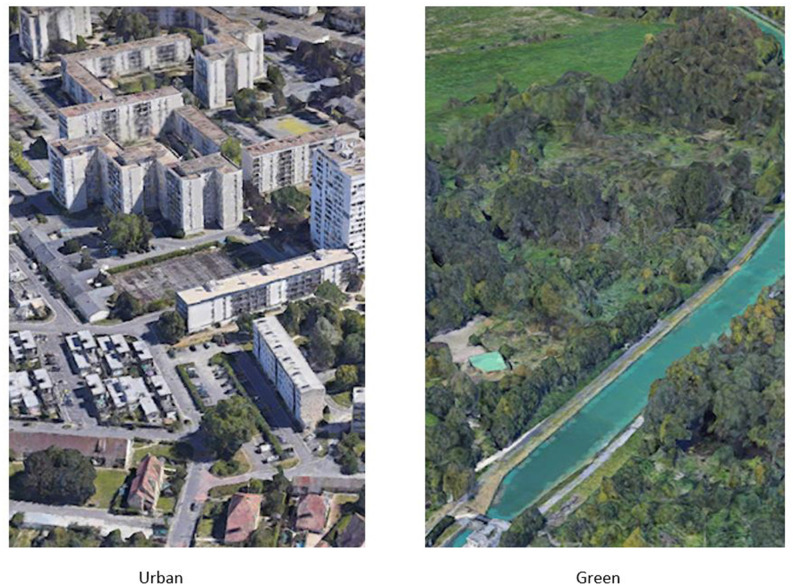
Satellite images of exposure environments (Urban, Green).

Participants in the green walking group (GW, *n* = 50) were asked to walk briskly for 30 min as if they were late for class. The walking sessions all occurred at the beginning of the afternoon (between 2:00 p.m. and 4:00 p.m.), were conducted outdoors, on a walkway alongside a canal, and were led by the first author. This walkway was lined with trees/bushes and passed through a mix of wine-growing areas, parklands, as well as wide open greenfields. Sessions were conducted in subgroups of 12–13 participants, which required the exercise bout to be repeated on four separate days to get through the whole group. Weather was quite similar during the testing days and quite normal for the season in Northeastern France; that is around 7°C–9°C, with no precipitation, and moderately cloudy conditions. The physical exercise stimulation was administered with the same parameters (type, duration, intensity, mode of delivery, subgroups size, time of day, and weather conditions) for participants in the urban walking group (UW, *n* = 50). However, walking sessions were conducted in a metropolitan urbanized district consisting of residential developments, commercial and nonresidential buildings, light industry, car parks, and totally asphalted streets. Figure 1 shows satellite images of the two exposure environments (green and urban) used in the present study.

On the contrary, the control group consisted of students from the same department at the same university who did not walk but attended a regular class lecture (CTRL, *n* = 50). This group involved two classes of 25 students randomly selected from a total of nine classes of the same year group at this department. For these two classes, the lecture took place at the same time of day as the walking sessions planned for participants in the UW and GW groups (between 2:00 p.m. and 4:00 p.m.). The lecture lasted 60 min and was divided into two 30-min modules; the first one was on the mental health benefits of outdoor physical activity, and the second one expanded on ideas presented right before toward the development of low-cost and effective nature-based interventions. Participants in both groups took the PANAS before and after walking (or before and after the first 30-min lecture module for those in the CTRL group). Heart rate was assessed *via* self-palpation of the radial pulse on the nondominant hand at the end of the walking sessions in the GW and UW groups.

### Data Analysis

A mixed design with two independent variables was used: (a) one between-participant variable (GW, UW, and CTRL groups), and (b) one within-participant variable (pre- and post-intervention assessments). There were two dependent variables: (a) Positive Affect (PA), and (b) Negative Affect (NA). Therefore, two 3 (Groups) × 2 (Time Points) mixed-design ANOVAs were conducted. The significance level was *α* = 0.05. Since we used multiple comparisons in *post-hoc* tests, the level of significance was partly modified by a Bonferroni correction to minimize the familywise error rate and maintain statistical power (Vincent, 2005). Effect sizes, Cohen’s *d* = (*Mi*–*Mj*)/*SD_pooled_*, were computed to compare the magnitude of PA and NA changes between participants who walked in connection to Nature and those who walked in the urbanized district. One-way ANOVAs and Chi-2 tests were also performed to assess whether there were baseline differences between groups. Finally, an independent sample Student’ *t*-test was used to compare the mean heart rate recorded at the end of the walking session for participants in both the GW and UW groups.

## Results

### Preliminary Analyses

**Figure 2:**
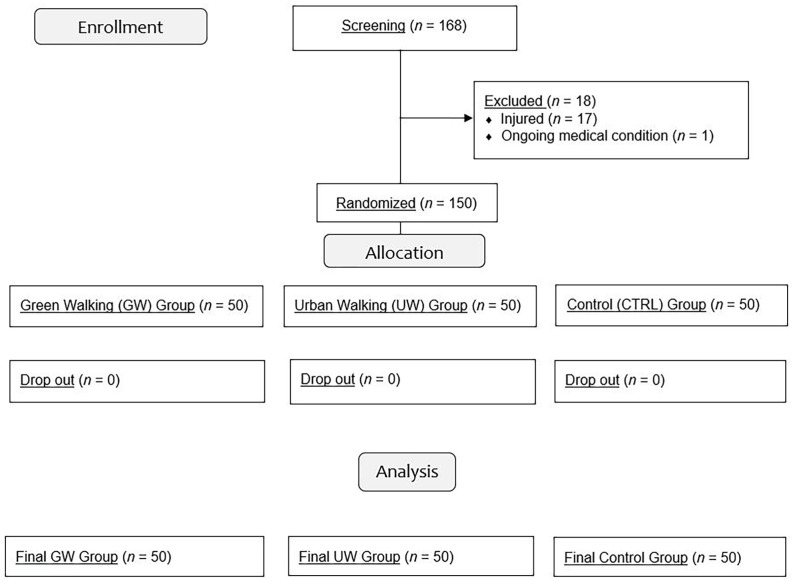
Flowchart of study participants through the trial.

All participants provided data pre- and post-intervention (see Figure 2). There were no differences between groups neither in terms of gender distribution χ^²^_(2)_ = 3.16, *p* = 0.207, nor in terms of age, *F*_(2,147)_ = 1.69, *p* = 0.188. Likewise, PA and NA scores did not significantly differ between groups at baseline ; *F*_(2,147)_ = 2.69, *p* = 0.072 and *F*_(2,147)_ = 2.91, *p* = 0.064 respectively. Lastly, heart rate at the end of the walking session in the GW group [*M* = 112.6 bpm ; that is 58.3% of age-predicted maximal heart rate (MHR)] was found to be statistically identical to that reported in the UW group (*M* = 111.2 bpm; i.e., 57.6% of age-predicted MHR), *t*_(98)_ = −0.38, *p* = 0.706. These values reflect physical exercise performed at «moderate» (55%–69% of age-predicted MHR) intensity according to the American College of Sports Medicine (Pollock et al., 1998).

### Positive Affect and Negative Affect

Descriptive statistics for the pretest and posttest PA and NA scores are given in Table 1. As can be seen, the mixed ANOVAs revealed significant Time × Group interaction for both variables. With regard to Positive Affect (PA), *post-hoc* analyses indicated that the mean PA score significantly increased from pre- to posttest for participants in the GW group (*t*_(49)_ = 3.93, *p* = 0.002) whereas it significantly decreased for participants who did not walk (CTRL group, *t*_(49)_ = −3.50, *p* = 0.009). Importantly, PA was not found to change significantly from pre- to post-walking among participants who exercised in the urban setting, *t*_(49)_ = 2.03, *p* = 0.661. The effect size comparing PA change in the two walking groups (i.e., GW *vs*. UW) was large, Cohen’s *d* = 0.83.

**Table 1:** Descriptive statistics for positive and negative affect scores as a function of Group and Time, ANOVA results, and effect sizes of the differences between the two intervention groups (Green Exercise vs. Urban Exercise) on the change score.

	Pretest		Posttest		*F*_(2,147)_	Cohen’s *d* [95% CI]
	*M*	*SD*	*M*	*SD*	Time × Group	
**Positive Affect (PA)**					15.54**	0.83 [0.42, 1.23]
GW group (*n* = 50)	29.90	5.84	32.58	6.18^†^		
UW group (*n* = 50)	31.60	6.82	30.22	7.16		
CTRL group (*n* = 50)	28.38	5.94	26.00	6.26^†^		
**Negative Affect (NA)**					6.56*	−0.11 [−0.57, 0.28]
GWgroup (*n* = 50)	16.50	5.28	12.56	2.60^†^		
UWgroup (*n* = 50)	17.18	6.50	13.62	3.65^†^		
CTRL group (*n* = 50)	14.70	3.81	13.60	3.25		

GW, Green Walking; UW, Urban Walking; CTRL, control. *p ≤ 0.01; **p ≤ 0.001; ^†^significant within-group score change from pretest to posttest (*post-hoc* Bonferroni-corrected tests).

Results for Negative Affect were quite different. More specifically, follow-up pairwise comparisons demonstrated that, regardless of the environment conditions, walking for 30 min at moderate intensity significantly reduced NA (GW: *t*_(49)_ = −6.54, *p* < 0.001; UW: *t*_(49)_ = −5.91, *p* < 0.001). On the other hand, NA remained statistically unchanged for participants in the control group, *t*_(49)_ = −1.83, *p* = 0.995. The effect size comparing NA change in the two walking groups was negligible, Cohen’s *d* = −0.11.

## Discussion

This study sought to directly compare the emotional effects of 30 min of outdoor walking in a green vs. urbanized setting. We found that green exercise significantly improved Positive Affect and significantly reduced Negative Affect, whereas the same exercise stimulus in a metropolitan urbanized area only decreased Negative Affect.

The finding that Positive Affect increased in the green but not in the urban walking group supports our research hypothesis and is in line with the findings from Hartig et al. (2003) or Park et al. (2011). However, several differences limit the comparability of results: (a) Hartig et al. (2003) asked half of their participants to perform various cognitive tasks before walking (attentional and memory tests); (b) exercise intensity was neither measured nor controlled in these two previous studies; (c) positive affect was measured using a relatively crude and nonspecific instrument in Hartig et al.’s (2003) study (the Zuckerman’s Inventory of Personal Reactions; ZIPERS, Zuckerman, 1977); and (d) there was no sedentary control group in Hartig et al.’s (2003) study.

Several mechanisms may be proposed to account for enhanced affective benefits during and following green exercise. First, air quality is one of the mechanisms outlined in Hartig et al.’s (2014) model of the links between Nature environments and well-being. As discussed by Zhang et al. (2017), both short and long-term exposures to polluted urban air, and particularly to fine particulate matter of aerodynamic diameter ≤2.5 μm (PM_2.5_), reduce subjective well-being and increase the rate of depressive symptoms. There is growing evidence from both toxicological and clinical studies that short-duration exposure to combustion-derived particles leads to immediate detrimental affective changes (World Health Organization, 2013). Reviews of the research literature report that vegetation decreases levels of air pollution by enhancing the removal of air pollutants through adherence to plant surfaces (adsorption) and/or absorption by leaf stomata (Anderson and Gough, 2021). Connecting with Nature *via* physical activity can serve as an alternative to polluted urban air. A second explanation might be that Nature immersion undergone during the green walking session contributed to the satisfaction of the need for nature relatedness which, according to Baxter and Pelletier (2019), should be viewed as an additional basic human psychological need. A fundamental tenet of the Self Determination Theory (SDT; Ryan and Deci, 2017) is that a direct relationship exists between basic psychological needs satisfaction and psychological well-being. Studies undertaken in various sports and physical activities (e.g., Gagné et al., 2003 in a sample of gymnasts; Quested and Duda, 2010 in a sample of dancers) tend to support SDT hypotheses regarding the satisfaction of basic needs as a predictor of performers’ well-being (i.e., self-esteem, positive affect). Finally, one of the classic explanations arising from environmental psychology, Attention Restoration Theory (ART; Kaplan, 1995), suggests that one’s affective state is linked to attentional resources, and defines two types of attention: directed and involuntary attention. Directed attention involves mental effort and concentration and, if overused, leads to mental fatigue and worsened affective state. Natural environments have been shown to promote the use of involuntary attention, providing an opportunity for recovery from mental fatigue, which is associated with positive change in affect (e.g., Faber-Taylor and Kuo, 2009).

By showing that green exercise has especially positive effects on emotional well-being that go beyond the benefits of being physically active, the present study identifies the various opportunities that outdoor sports can offer in order to help prevent or alleviate mental health problems. Some research teams have provided preliminary data suggesting that physical activity in a natural environment might produce greater mental health benefits than physical activity elsewhere (e.g., Mitchell, 2013). The large cross-sectional health survey by Mitchell (2013) revealed that the odds of poor mental health among those regularly using woods or forests for physical activity were almost 50% lower (OR = 0.56) compared to non-users. However, considerably more research is needed to establish the therapeutic benefits of green exercise in the treatment of mental disorders for which low positive affect is a prevalent feature (i.e., depressive disorders).

Our findings come with some limitations. First, future research should aim at increasing the generalisation of green exercise practice, using a wider variety of social groups while exercising, and involving various degrees of «naturalness». Future research should also consider assessing the impact of green exercise on a wider range of psychological outcomes (stress, anger-hostility) and with different population samples (non-regular exercisers, indoor exercisers, highly depressed individuals, and individuals raised in non-urban areas). The sample in the current study consisted of young people who were engaging regularly in physical activities (including activities in the wilderness such as mountain-biking and kayaking) due to their curriculum, and were thus likely to be relatively healthy and positively oriented towards Nature. Finally, the present study focused on acute exercise effects, but it may be advantageous to study the effects of longer-term green exercise programs, particularly if exercise is aimed at individuals with depressive symptoms. Indeed, available recommendations regarding optimal exercise «dosages» based on research including individuals with clinical depression state that exercise interventions should last at least 10 weeks (Rethorst and Trivedi, 2013).

## Conclusion

In conclusion, despite the above-mentioned limitations, our findings have implications for public mental health and deserve the emerging discussion surrounding ecosystem services (Hughes et al., 2013). In addition, greater awareness of the indirect benefits to humans of natural environments could help contribute to their protection and maintenance.

## Data Availability Statement

The raw data supporting the conclusions of this article will be made available by the authors, without undue reservation.

## Ethics Statement

The studies involving human participants were reviewed and approved by Comité d’Ethique de Recherche en Sciences et Techniques des Activités Physiques et Sportives (CER STAPS). The patients/participants provided their written informed consent to participate in this study.

## Author Contributions

FL and PJ contributed to the conception and design of the study and drafted the manuscript. FL and GP acquired the data and performed the data analysis. FL, FB, and PJ edited and revised the manuscript. All authors interpreted the results, read, and approved the final version of the manuscript. All authors contributed to the article and approved the submitted version.

## Conflict of Interest

The authors declare that the research was conducted in the absence of any commercial or financial relationships that could be construed as a potential conflict of interest.

## Publisher’s Note

All claims expressed in this article are solely those of the authors and do not necessarily represent those of their affiliated organizations, or those of the publisher, the editors and the reviewers. Any product that may be evaluated in this article, or claim that may be made by its manufacturer, is not guaranteed or endorsed by the publisher.
